# Perceptual Learning of Interrupted Speech

**DOI:** 10.1371/journal.pone.0058149

**Published:** 2013-03-01

**Authors:** Michel Ruben Benard, Deniz Başkent

**Affiliations:** 1 Pento Audiology Center Zwolle, Zwolle, The Netherlands; 2 University of Groningen, University Medical Center Groningen, Department of Otorhinolaryngology/Head and Neck Surgery, Groningen, The Netherlands; 3 University of Groningen, Graduate School of Medical Sciences, Research School of Behavioral and Cognitive Neurosciences, Groningen, The Netherlands; UNLV, United States of America

## Abstract

The intelligibility of periodically interrupted speech improves once the silent gaps are filled with noise bursts. This improvement has been attributed to phonemic restoration, a top-down repair mechanism that helps intelligibility of degraded speech in daily life. Two hypotheses were investigated using perceptual learning of interrupted speech. If different cognitive processes played a role in restoring interrupted speech with and without filler noise, the two forms of speech would be learned at different rates and with different perceived mental effort. If the restoration benefit were an artificial outcome of using the ecologically invalid stimulus of speech with silent gaps, this benefit would diminish with training. Two groups of normal-hearing listeners were trained, one with interrupted sentences with the filler noise, and the other without. Feedback was provided with the auditory playback of the unprocessed and processed sentences, as well as the visual display of the sentence text. Training increased the overall performance significantly, however restoration benefit did not diminish. The increase in intelligibility and the decrease in perceived mental effort were relatively similar between the groups, implying similar cognitive mechanisms for the restoration of the two types of interruptions. Training effects were generalizable, as both groups improved their performance also with the other form of speech than that they were trained with, and retainable. Due to null results and relatively small number of participants (10 per group), further research is needed to more confidently draw conclusions. Nevertheless, training with interrupted speech seems to be effective, stimulating participants to more actively and efficiently use the top-down restoration. This finding further implies the potential of this training approach as a rehabilitative tool for hearing-impaired/elderly populations.

## Introduction

Normal-hearing listeners use several top-down mechanisms that help speech perception in difficult listening environments. They may, for example, perceptually restore inaudible or masked portions of temporally interrupted speech, taking advantage of the context and redundancy in speech signals, as well as using linguistic rules, prior knowledge, and expectations [1]–[4]. In the special case of phonemic restoration, the restoration benefit is commonly shown by the increase in intelligibility of periodically interrupted speech when the silent intervals are filled with noise bursts that would be capable of masking the speech [2]–[9]. Increase in intelligibility as a result of adding noise to speech signals is somewhat counterintuitive. However, in the case of restoration, the filler noise adds ambiguity for the perceptual system, where the system then tends towards forming a full object, rather than perceiving the individual pieces per se, referred to as the Gestalt principles of closure [2], [3]. These closure mechanisms, then, presumably help with the speech restoration.

A number of hypotheses have been proposed to explain the underlying mechanisms of restoration that produce the improvement in intelligibility with the filler noise. Huggins [10] noted that the filler noise masks the distortions that occur due to the sudden onsets and offsets in interrupted speech, and thus suggested that the bottom-up processes of the auditory system are entirely responsible for this benefit. Others, on the contrary, pointed to the involvement of the high-level cognitive processes, based on the influence that the context and the type of speech materials used had on the perception of interrupted speech [5], [8], [11]–[15]. Recent studies that showed a deficit in restoration benefit with (real or simulated) hearing impairment implied that the restoration may actually be governed by a combination of the bottom-up peripheral and top-down cognitive processes [16]–[22], in agreement with general high-level speech and sound perception mechanisms in complex listening environments [23]–[28]. Hence, the consensus from recent studies is that cognitive processes are involved in the phonemic restoration mechanism, but up to what degree is still not clear.

Based on their observations, Verschuure and Brocaar [7] suggested that the degree of involvement of cognitive processes might differ in the perception of interrupted speech with silent intervals from the perception of interrupted speech combined with filler noise. For example, without the filler noise, the listeners were anecdotally reported to be aware of the silent intervals in the signal, and seemed to be forced to guess consciously what could have been presented to them. With the noise, the listeners unconsciously filled in the missing speech information. One could expect different effects of training on tasks that require different cognitive resources and that differ in how automatic and effortless they are [29]. Therefore, exploring learning effects with interrupted speech with or without the filler noise could be used to show if there is such a difference.

Other than indicating potentially varying cognitive processes, perceptual learning effects could reveal other factors relevant to phonemic restoration. Interrupted speech with silent intervals is a less ecologically valid signal than interrupted speech with filler noise, because in real life speech is more often obliterated by noise than by silence. A difference in intelligibility may then be observed between the two forms of interruptions, not due to the restoration benefit per se, but due to the participants being less used to hearing such artificial manipulations. If the restoration benefit of adding filler noise were not a real effect but a consequence of such an artifact, then it would be expected to diminish or disappear after listeners are exposed to and trained with these artificial speech stimuli.

The present study explored the effects of perceptual learning, more specifically the improvement in performance after systematic long-term training [30]–[32], on the perception of interrupted speech. The purpose was to explore the hypotheses that the cognitive involvement could differ for understanding interrupted speech with or without the filler noise, and that the restoration benefit could be an artifact of using interrupted speech with silent intervals, an ecologically not valid signal produced by an artificial manipulation. Participants were systematically trained with speech manipulated with two kinds of interruptions, with silent intervals or filler noise, and speech intelligibility and perceived mental effort were measured before, during, and after training. The training part was designed based on previous studies on perceptual learning. The performance on many auditory skills improves with training [30], [31], [33], commonly given in the form of an explicit training [34], although improvement due to unattended exposure is also possible [35]. While humans adapt relatively automatically to rather simple stimuli [36], more complex ones, such as speech manipulated with time compression [37], spectral reduction [38], [39], or interruptions [40], may need more effort to adapt to. Based on the studies listed above and due to the complex nature of the stimuli, an intensive training with feedback was preferred. If the cognitive involvement varied between the two kinds of speech signals, the effort requirement of the two tasks and the effects of learning on intelligibility and perceived effort would be expected to differ. If the restoration benefit were due to an artifact of using interrupted speech with silent intervals, it would be expected to diminish or disappear at the end of training.

## Materials and Methods

### A. Listeners

Thirty normal-hearing listeners, ages between 18 and 28 years (M_age_ = 21.3 years, SD = 2.4 years, 21 women), participated in the study. During the initial screening, normal hearing via a hearing test (at test frequencies of 0.5 kHz up to 4 kHz, hearing thresholds of 20 dB HL or less) and normal development of speech and language via a questionnaire were confirmed. The listeners, all native speakers of Dutch, were divided into three groups, matched on age and gender. The baseline performances were measured before and after the training sessions. Two groups received training with feedback between the baseline measurements. The noise group (NG) was trained with interrupted speech with the filler noise and the silence group (SG) without. The third group did not receive any training. They were only tested with baseline conditions applied at two different days, and thus served as the control group (CG). From the SG and the NG, 7 and 6 listeners, respectively, participated in a follow-up testing at a later time to observe the retainability of the learning effects.

### B. Ethics Statement

The study was approved by the Medical Ethical Committee of the University Medical Center of Groningen. The listeners were recruited by poster announcements at public places and participation was compensated financially. Information about the experiment was provided and written informed consent was collected prior to participation.

### C. Stimuli

The speech stimuli were Dutch sentences digitally recorded at 44.1 kHz sampling rate and spoken by a male speaker [41]. The sentences are semantically neutral and represent conversational speech. The database consists of 39 sets. Each set contains 13 sentences, with 4 to 9 words per sentence, and 74 to 88 words in total. The sentences were interrupted by a cosine-ramped (ramp duration of 10 ms) periodic square wave with 1.5 Hz interruption rate and 50% duty cycle. This resulted in speech portions followed by interruptions of 333 ms of duration each. Former studies [6], [11], [17], [21], [22] and our pilot study have shown that these parameters produced low baseline intelligibility of interrupted speech with silent gaps. Thus, there was ample room for potential improvement in intelligibility after both adding the filler noise (restoration benefit) and training the listeners (perceptual learning). The noise used as filler was the steady speech-shaped noise generated by Versfeld et al. [41], that matched the long-term average speech spectrum of the recorded sentences. The filler noise bursts were produced by applying the same periodic square wave, except with inverted phase, to the speech-shaped noise. The interrupted speech and the noise bursts were combined in a way such that there was sufficient but minimal overlap between the two, with no apparent change in overall energy during the transitions (see [9] for details).

The root mean square intensity was normalized to the same fixed value for all sentences. The presentation levels of the speech and the filler noise were calibrated to 60 and 70 dB SPL (based on [21]), respectively, when measured at an approximate position of the participant’s head.

### D. Experimental Procedure

The participants were seated in an anechoic chamber, facing the free-field loudspeaker and the monitor that presented the visual feedback at a distance of 1 m. The digitized processed stimuli were directed from an external AudioFire 4 soundcard of Echo Digital Audio Corporation to a Tannoy 8D Precision active near-field speaker. The experimenter was seated outside the anechoic chamber and listened to participants’ responses via a headphone connected to the digital voice recorder, DR-100 digital by Tascam, of the anechoic room. As the stimuli were presented in free field, the experimenter inadvertently also heard the stimuli. Any potential bias that may have been caused by this single-blind design must have been negligible, as the restoration effect observed in the present study was comparable to the restoration effects observed in double-blind versions conducted by our research group [21].

The experimental procedure consisted of initial and final baseline measurements of intelligibility and perceived effort, with training sessions in-between (Table 1; details below). The difference in the baseline scores before and after training thus showed the improvement in performance due to perceptual learning as a result of training. The interval between the initial and final baseline measurements varied slightly, between 2 and 3 days, depending on the availability of the participants. The training was spread over three days and the entire experiment, including the participant screening and initial and final baseline tests, was completed in less than one week. At a later time, 6 to 18 weeks after the training was completed, a follow-up baseline test was conducted to observe how the training effects were retained. In the entire study, we used a MATLAB program to process the stimuli online and to present the processed stimuli and audio and visual feedback to the participants via a graphical user interface.

**Table 1:** Experimental procedure, shown for the noise (NG), silence (SG) and control (CG) groups, along with the number of participants (n).

Groups	Baseline measurement before	Training	Baseline measurementafter	Follow-up baseline measurement
SG (n = 10)	Silence and noise	Five silence training sessions	Silence and noise	(n = 7), silence and noise
NG (n = 10)	Silence and noise	Five noise training sessions	Silence and noise	(n = 6), silence and noise
CG (n = 10)	Silence and noise	No training or testing session	Silence and noise	

“Silence” denotes testing with interrupted sentences with silent intervals, and “noise” denotes testing with interrupted sentences that are combined with filler noise bursts. The CG did not receive training; they were only tested with the baseline measurements at two different times, with an in-between time comparable to that of the training duration.

In the baseline measurements before and after training, all three groups were tested on speech intelligibility and perceived mental effort with interrupted speech, with and without the filler noise. In each condition of the baseline measurements, 2 sets (26 sentences) were randomly selected from the 39 sets. As a result, participants were exposed to 52 unique sentences before and 52 unique sentences after training. In baseline measurements and during training, no sentence was heard more than once. In the intelligibility tests, the participants listened to one sentence at a time, and they were instructed to repeat all of the words they heard, even if this led to nonsense sentences. Guessing the missing words was encouraged as the purpose of the test was to assess the reconstructed perception of the sentence, rather than what is heard per se. The participants were instructed to tell the experimenter when they were ready for the next sentence (by saying *Next*). Scoring of correctly repeated words was first performed in real time by the experimenter, and was later double-checked by offline listening to digital recordings of participants’ responses. All words were included in the scoring. The percentage of correctly identified words was calculated as the ratio of the total number of correctly repeated words to the total number of words within the sets. The participants heard a set of sentences only once, and they were not familiar either with the speech material used or with listening to interrupted speech in general before their participation in this study.

During the training sessions, as shown in Table 1, the NG and SG were trained with different stimuli. The CG received no training, nor did they attend the training sessions. They participated only in two sessions of baseline measurements. The duration between the two sessions was comparable to the duration between the two baseline measurements (before and after training) of the trained groups. In each of the 5 training sessions, 26 sentences were used, so that the SG and NG were trained with 130 sentences. Hence, in total, the CG was exposed to 104 unique sentences and the SG and NG to 234 unique sentences. The difference in the training sessions compared to the baseline measurement sessions was that during the training feedback was provided. After receiving the participant’s response, first the unprocessed then the processed sentence were played back (based on [33]), while the text of the sentence was simultaneously displayed on the computer screen.

At the end of each session (baseline or training) and for all participants, the perceived mental effort was measured using the Visual Analogue Scale (VAS), a subjective measure shown to be sensitive to small differences in mental effort [42], [43]. This method, while not evaluated objectively in previous studies, was selected due to the ease of use. The participants were instructed to rate the effort of the comprehension for the entire session by a mark on a 10 cm long scale, varying from “effortless” (0 on VAS-scale) to “effortful” (10 on VAS-scale) on paper. Listening to a known poem in quiet (effortless) and having a conversation in loud noise (effortful) were given as examples to the participants to interpret the full range of the VAS scale.

## Results

### A. Speech Intelligibility

**Figure 1:**
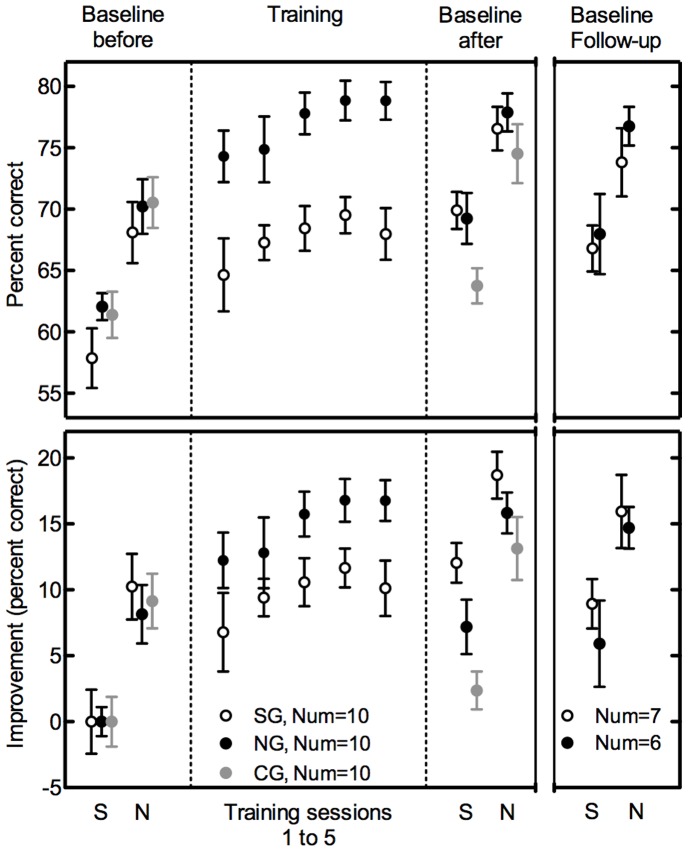
Intelligibility of interrupted speech with and without filler noise. The absolute mean percent correct scores from all listener groups are shown for baseline and training sessions in the top panel. The relative mean percent correct improvement, calculated by normalizing the absolute scores with respect to the ‘S’ condition before training, is shown in the bottom panel, The ‘S’ (Silence) and ‘N’ (Noise) on the horizontal axes denote the conditions with interrupted sentences with silent intervals and with filler noise in the interruptions, respectively. The open, filled, and gray data points represent the results from the silence (SG), noise (NG), and control (CG) groups, respectively. The panels from left to right show the results of baseline measurements before training, measurements made right after each training session during the training, baseline measurements after training, and the follow-up baseline measurements conducted at a later time (also see Table 1). The CG received no training and were only tested with the baseline measurements. Error bars denote one standard error of the mean.

The top panel of Fig. 1 shows the mean percent correct scores for all sessions (baseline and training), as well as the follow-up baseline measurement; the bottom panel shows the increase in percent correct for all sessions, relative to the silence (S) condition of the baseline measurement before training. The purpose of the normalization in the lower panel was to better visualize the change in intelligibility due to training, as well as due to the addition of the filler noise. The baseline speech intelligibility scores measured before and after the training are shown in the first and third segments of Fig. 1, respectively, in both top and bottom panels (also summarized in Table 2). These data show that there was a restoration benefit before training with each listener group, and even though the training increased the scores in both S and noise (N) conditions, a similar restoration benefit could still be observed after the training. In the initial baseline measurement, on average, there was a restoration benefit of 9.2%, as shown by the increase in scores with the addition of the filler noise (‘N’ column compared to the ‘S’ column in “before training” scores in Table 2). After the training, a similar restoration benefit was observed with, on average, 8.7% (middle column of Table 2). Repeated measures ANOVAs were performed with both forms of the percent correct scores, the absolute percent correct scores in Fig. 1, top panel, and the relative percent correct scores in Fig. 1, bottom panel, with addition of filler noise and training as within-subjects factors and participant group as the between-subjects factor. The ANOVAs showed that this restoration benefit was significant (F(1,27) = 106.4, p<0.001, partial η^2^ = 0.798, power = 1). The improvement after the training sessions is shown in the increase of scores in S and N conditions from before to after baseline measurements in Fig. 1, and also in the rightmost columns of Table 2. The training produced significant overall improvement (F(1,27) = 28.3, p<0.001, partial η^2^ = 0.512, power = 1), varying from 7.2 to 12%, for both training groups and for both testing conditions of S and N. Although the CG improved in performance as well, their improvement was smaller, 2.4% to 4%. The analysis performed with the absolute percent correct scores (top panel) showed no significant difference between the three groups (F(2,27) = 1.2, p = 0.307, partial η^2^ = 0.084, power = 0.25) and no significant interaction effect. The analysis performed with the relative percent correct scores (bottom panel), however, showed a significant difference between the three groups (F(2,27) = 3.6, p = 0.041, partial η^2^ = 0.211, power = 0.62) and no significant interaction effect. Note that the SG started from a lower baseline performance level than the NG and CG (Fig. 1, top panel). Hence, the training effect was highest for the SG (Fig. 1, lower panel).

**Table 2:** The absolute (top rows) and relative (bottom rows) mean percent correct (PC) scores of the baseline measurements before and after training of the SG and NG (left and middle columns), and overall improvement taken from Fig. 1 (right column).

Groups	Absolute PC scores baseline before (%)		Absolute PC scores baseline after (%)		Improvement (%)	
	S	N	S	N	S	N
SG (n = 10)	57.9	68.1	69.9	76.6	12.0	8.5
NG (n = 10)	62.1	70.2	69.2	77.9	7.2	7.7
CG (n = 10)	61.4	70.5	63.8	74.5	2.4	4.0
	**Relative PC scores baseline before (%)**		**Relative PC scores baseline after (%)**		**Improvement (%)**	
	**S**	**N**	**S**	**N**	**S**	**N**
SG (n = 10)	0.0	10.2	12.0	18.7	12.0	8.5
NG (n = 10)	0.0	8.1	7.2	15.8	7.2	7.7
CG (n = 10)	0.0	9.1	2.4	13.1	2.4	4.0

The CG received no training and were only tested with the baseline measurements, to see the potential learning effects due to the exposure to testing paradigm only, in the lack of a targeted training. ‘S’ and ‘N’ refer to testing conditions with interrupted sentences with silent intervals or with filler noise, respectively.

The middle section of the top panel of Fig. 1 shows the absolute percent correct scores measured during the training sessions where the feedback was provided; the bottom panel shows the same, except that the scores are normalized relative to the S condition of the baseline measurement before training. These data show that training increased the scores with interrupted speech with or without the filler noise, but the intelligibility of interrupted sentences combined with filler noise was always higher than the interrupted sentences with silent intervals. This means that the restoration benefit observed in the baseline measurement before the training was retained throughout the training. Repeated measures ANOVAs were performed with both absolute and relative percent correct scores, with the training sessions as within-subjects factor and the addition of filler noise as between-subject factor. These showed that the improvement in both absolute and relative scores between the five training sessions was not significant (F(4,15) = 2.0, p = 0.145, partial η^2^ = 0.106, power = 0.47). The same was true for both absolute and relative scores, as the normalization did not change this effect. But the restoration benefit due to added noise occurred for both absolute (F(1,18) = 35.9, p<0.001, partial η^2^ = 0.667, power = 1) and relative percent correct scores (F(1,18) = 10.9, p = 0.004, partial η^2^ = 0.377, power = 0.88). There was no significant interaction effect.

The right segments of Fig. 1 show the intelligibility of the follow-up testing, performed with 7 participants from the SG and 6 participants from the NG, at 42 to 127 days (M = 92 days, SD = 27 days) after the second baseline measurement. These data show that the restoration benefit was still significant, and overall, the scores were more similar to the trained-level scores than the initial un-trained level scores. Repeated measures ANOVAs were conducted on this subset of listeners only, with the within-subjects factors of testing time (after training or follow-up) and adding the filler noise, and the between-subjects factor of the participant group. There was no significant effect of testing time (F(1,11) = 1.4, p = 0.265, partial η^2^ = 0.111, power = 0.19), but a significant effect of restoration benefit (F(1,11) = 23.6, p = 0.001, partial η^2^ = 0.682, power = 0.99), for both representations of data. There was no significant interaction effect. The analysis performed with the absolute percent correct scores (F(1,11) = 0.04, p = 0.853, partial η^2^ = 0.003, power = 0.05, top panel) and with the relative percent correct scores (F(1,11) = 3.2, p = 0.101, partial η^2^ = 0.226, power = 0.37, bottom panel) showed no significant difference between the two groups.

### B. Perceived Mental Effort

**Figure 2:**
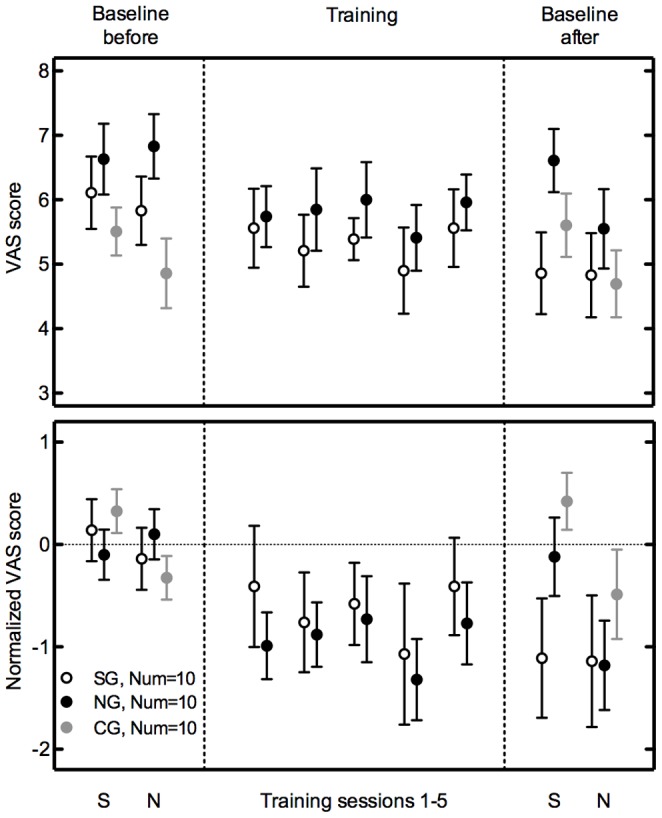
Perceived mental effort. The absolute and normalized mean mental effort scores are shown in the top and bottom panels, respectively. These scores are measured by means of a visual-analogue scale (VAS), varying from “effortless” (0 on VAS-scale) to “effortful” (10 on VAS-scale). The first and the third panels show the scores measured before and after the training, respectively. The middle panel shows the scores during the training sessions. Error bars denote one standard error of the mean.

The top panel of Fig. 2 shows the mean perceived mental effort scores for all testing sessions, and the bottom panel shows the mean perceived mental effort scores normalized over the average of the S and N conditions before training. The purpose of this normalization, different than Fig. 1, was to minimize the variability in the utilization of the VAS-scale between the participants. Therefore, the scores were not normalized with respect to ‘S’, but instead, with respect to participants’ own baseline ratings. The first and third segments of both panels of Fig. 2 represent the perceived mental effort of the baseline measurements before and after the training sessions, respectively. These data show that while there was a tendency for the N condition to be perceived less effortful compared to the S condition, during and after the training sessions, there were also some exceptions, such as the S condition after the training. In the initial and final baseline measurements, there was on average a significant decrease in perceived mental effort with the addition of the filler noise (‘N’ column compared to the ‘S’ column in “before training” and “after training” scores in Table 3; F(1,27) = 7.0, p = 0.014, partial η^2^ = 0.205, power = 0.72, for both absolute and normalized VAS-scores). The training significantly reduced the perceived mental effort, shown by the decrease in VAS between “before” and “after” baseline measurements in Fig. 2, and also in the rightmost columns of Table 3 (F(1,27) = 8.0, p = 0.009, partial η^2^ = 0.228, power = 0.78, for both representations of the VAS-scores). There was no significant difference between the three groups both when represented in absolute (F(2,27) = 2.35, p = 0.114, partial η^2^ = 0.148, power = 0.43) and normalized VAS-scores (F(2,27) = 2.20, p = 0.130, partial η^2^ = 0.140, power = 0.41). There was no significant interaction effect.

**Table 3:** Similar to Table 2, except the scores shown are the absolute mean perceived mental effort scores (top rows) and the normalized mean perceived mental effort scores with respect to the baseline measurements before training (bottom rows), measured by means of a visual-analogue scale (VAS).

Groups	VAS scores before		VAS scores after		Improvement	
	S	N	S	N	S	N
SG (n = 10)	6.1	5.8	4.9	4.8	1.3	1.0
NG (n = 10)	6.6	6.8	6.6	5.6	0.0	1.3
CG (n = 10)	5.5	4.9	5.6	4.7	−0.1	0.2
	**Normalized VAS scores before**		**Normalized VAS scores after**		**Improvement**	
	**S**	**N**	**S**	**N**	**S**	**N**
SG (n = 10)	0.14	−0.14	−1.11	−1.14	1.25	1.00
NG (n = 10)	−0.10	0.10	−0.12	−1.18	0.02	1.28
CG (n = 10)	0.33	−0.33	0.42	−0.49	0.09	0.16

The middle sections of Fig. 2 show the VAS-scores measured during the training, after each training session. These data show that there was no significant change in VAS scores during the training (F(4,15) = 1.4, p = 0.269, partial η^2^ = 0.049, power = 0.34, for both absolute and normalized VAS-scores). The absolute VAS-scores show that the SG rated the perceived effort lower than the NG, but the difference was not significant (F(1,18) = 0.54, p = 0.472, partial η^2^ = 0.029, power = 0.11). This difference is also not significant for the normalized VAS-scores (F(1,18) = 0.37, p = 0.550, partial η^2^ = 0.020, power = 0.09). There was no significant interaction effect.

## Discussion

Before training, a baseline intelligibility of interrupted sentences, with and without the filler noise, was measured. These pre-training results were comparable to previous studies on intelligibility of interrupted speech [16], [22], [44], [45], and on restoration benefit observed with additional filler noise in silent intervals [5], [6], [7], [9].

The first interest of the present study was to observe the effect of training on the perception of interrupted speech with silent intervals and with the filler noise, as a way of exploring the similarity in the underlying cognitive mechanisms involved in the perception of the two types of stimuli (with or without the filler noise). Verschuure and Brocaar [7] hypothesized, based on their observations during their study, that the perception of speech interrupted by silence involves other cognitive processes than the perception of interrupted speech combined with filler noise. We further hypothesized that if the cognitive involvement varied in the perception of the two kinds of speech signals, they would be learned at different rates with training. The speech intelligibility results showed that the percent correct scores increased during the training sessions similarly for both training groups. In other words, speech with both forms of experimental manipulations could both be learned, and in a similar manner too. Hence, the results imply that speech perception with both forms of interruptions (with silence or with filler noise) involves similar cognitive mechanisms.

In addition to speech intelligibility, perceived mental effort was also measured. Processes requiring cognitive awareness are suggested to be more effortful than unconscious processes [46]. Based on the observations by Verschuure and Brocaar [7], therefore, we had hypothesized that if cognitive mechanisms differed between the perception of interrupted speech with or without the filler noise, we would see a difference in the perceived effort scores with the two forms of speech. In fact, the analysis of the perceived mental effort showed on average a small, though significant, decrease in VAS-score with the addition of the filler noise, both before and after training. However, because of irregularities in the scoring between the groups, such as the high score of the NG for the S condition after training, we reckon that these differences in VAS-scores, although normalized, stem from the individual preferences of different groups, rather than a direct result of the experimental manipulation. There was no systematic change in effort scores during training. However, when the scores were compared for before and after training, there was a decrease in the rating of the perceived mental effort, and in similar values for the two forms of interruptions. These results on perceived effort, hence, only partially support the hypothesis.

The second hypothesis of the study was that if the restoration benefit was due to an artifact of using interrupted speech with silent intervals, an unusual and less ecologically valid form of speech, it would be reduced or entirely disappear at the end of training. This idea was also suggested by Verschuure and Brocaar [7], who reported that participants did not benefit from adding noise in the silent intervals when they were familiar (i.e. trained) with this type of interrupted speech. The suggestion was only anecdotal, as their data were limited due to the ceiling effects and there was no systematic investigation of learning effects. The results from the present study are in contradiction to the observations by Verschuure and Brocaar [7], because training did not bring the intelligibility of the two forms of interrupted stimuli to the same level. By training the participants, we observed a relatively similarly increasing curves in the overall percent correct scores of the SG and NG, and the restoration benefit persevered. The baseline measurements after the training showed that the restoration benefit of adding noise was still present after training, indicating that the restoration benefit is not an effect due to the artificiality of interrupted speech with silent intervals.

During the training, a plateau was observed in the scores, in a similar manner between the two training groups. We interpreted this as that the groups reached the limit of learning with these stimuli and that sufficient training was given. We made our conclusions based on this interpretation. However, there were perhaps some additional factors that affected the results. For example, we cannot exclude the possibility that a part of the increase in performance can be explained by the familiarity of the participants with the talker’s voice [47], [48], as we used sentences spoken by one talker only. Because the SG and NG were trained with different stimuli, stimuli-specific effects might also have additionally (but perhaps only slightly) influenced the shape of the increasing curves of these groups. Further, null results combined with a relatively small number of subjects indicate that the paradigm used in the present study was perhaps not sufficient to fully validate the conclusions, and further research with more statistical power would be needed to more confidently make such conclusions.

The findings of the present study may have practical implications. The training results show a potential benefit of the specific training paradigm used in the study. Note that the amount of speech information provided and the distortions in the signals caused by interruptions were the same across training sessions, and yet, the intelligibility of interrupted speech, with or without the filler noise, increased significantly as a result of the training. This outcome not only suggests that the restorative mechanisms for understanding interrupted speech are probably highly cognitive, but also that our training paradigm seems to train the listeners effectively to make better use of the top-down repair mechanisms. The training was generalizable; participants showed an increase in performance also for the other speech manipulation than the one they were trained with. Additionally, the training effects were retained, in line with earlier perceptual-learning studies [49]–[52]; several weeks after the training the percent correct scores were not significantly different from the baseline measurements taken immediately after the training. Perceptual learning, a relatively permanent change of perception as a result of training [30], was hence achieved. These observations point to the potential benefits of the type of training used in the present study as a tool for speech perception rehabilitation, for example, for the (elderly) users of hearing aids and cochlear implants [53]–[57].
